# A Plea for Good Global Governance

**DOI:** 10.3389/fpubh.2015.00046

**Published:** 2015-03-10

**Authors:** Ulrich Laaser

**Affiliations:** ^1^Section of International Public Health, Faculty of Health Sciences, University of Bielefeld, Bielefeld, Germany

**Keywords:** globalization, governance, health, DAH, EPHF, global threats

We are facing a rapid globalization in the twenty-first century. This sharpens our view on this one planet (1) traveling through space, the Spaceship Earth. It is largely in our hands as a human race whether this journey is a safe one. However, we can summarize the global threats endangering us – the passengers – as shown in Figure 1.

**Figure 1:**
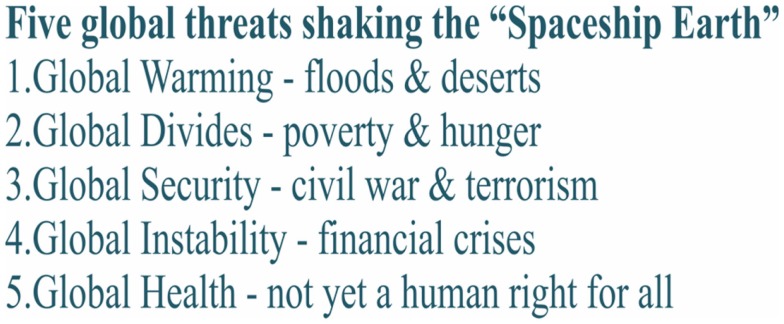
**Core global threats of twenty-first century**.

All five threats affect our health but cannot be controlled at the national level. They are the consequence of ineffective, failing, or even totally missing global governance. Certainly, the millennium development goals (MDGs) are partly successful in a global scale due mainly to the overachievement of China (2); however, many of the poorest countries are left out and the Ebola crisis developed not by chance in Sierra Leone and Liberia, countries torn by civil war, leaving the health services destructed (3).

Do we at least speak the same professional language? The answer is NO. Even the most basic so-called “Essential Public Health Functions” vary between WHO regions (2) as do the definitions of public health or population health. Recently, at the 14th World Congress of Public Health in Kolkata, India a first serious effort has been made to agree on common global public health functions (GPHF) (4) as there are three overlapping areas for action, namely, health promotion and protection together with prevention are embraced by four enabling and supporting functions, namely information, governance, advocacy, and capacity. A main purpose is to scale up workforce development in public health and to advance the investment case for public health promotion, protection, and prevention.

Is good global governance for health in reach? As it seems the answer is NO again. The main obstacle securing equitable “Health for All” today is posed by the five threats listed in Figure 1, not by any of the vertical disease programs competing globally for resources. A typical example of this is the much praised HIV/AIDS programs, which succeed to a large degree by draining the regular health services from the top staff by considerably higher remuneration.

The global government we need also cannot be based on a multiplicity of around 40 United Nations organizations and 25 development banks. Worse do the fragmentation and the lack of coordination and accountability in the sector of Non-Governmental Organizations (NGOs), comprising thousands of organizations, which represent about a quarter of total development assistance for health (DAH). Obligatory accreditation for NGOs therefore has been argued for (2).

So in conclusion: what to do? A global government will be there – 1 day. Hopefully, not too late! An interim step could be to further strengthen regional collaboration not only on a fragile voluntary basis but also organized as long-term binding agreements (5). Examples of a successful regionalization of transnational governance including the health sector are given by the European Union and ASEAN. Beyond that some key areas for advancing global governance can be identified (5):

(1) The mandate of the World Health Organization needs to be reconsidered in terms of an umbrella to include and coordinate all actions necessary to deal with the health consequences of the five global failures listed above.
(2) An enlarged mandate of WHO needs to be based on the inclusion of all stakeholders, private or public, beyond the present restriction to national governments.
(3) A systematic follow-up on the Monterrey and the Paris/Accra/Busan criteria, making development cooperation more effective.
(4) Consider proposals to improve national coordination by sector-wide approaches (SWAp).
(5) Transform innovative health and social care practices to education and training. Achieving a transformation of health systems that impact directly and effectively on the health and well-being globally depends on broadening opportunities for learning at all societal levels and across all nations.

Many questions wait for an answer from the global community, especially on a more democratic, effective, and efficient global governance for health or in other words: what global, regional, national and local structures, organizational principles, and mechanisms should ideally evolve in the early decades of this century to improve and sustain global health and well-being, including universal health coverage.

However, what is the likelihood that recommendations will be implemented successfully and impact positively and ethically on people’s lives? Progress has been slow and agonizingly much too late in many instances and calls for a new type of leadership, which transcends self-interests, is informed by long-term global people and planetary perspectives, and strives to make the quality of life and well-being of all people the top priority for public health.

## Conflict of Interest Statement

The author declares that the research was conducted in the absence of any commercial or financial relationships that could be construed as a potential conflict of interest.
